# Supplementary material for: A Global Screen for Assembly State Changes of the Mitotic Proteome by SEC-SWATH-MS
Source: Cell Syst. 2020 Feb 26;10(2):133–155.e6. doi: 10.1016/j.cels.2020.01.001 (PMC7042714; doi:10.1016/j.cels.2020.01.001)

Q9NZJ7 | MTCH1_HUMAN | MTCH1 PSAP CGI−64 UNQ1871/PRO4314
Monomer MW [kDa]: 41.544 Monomer expected elution fraction: 48

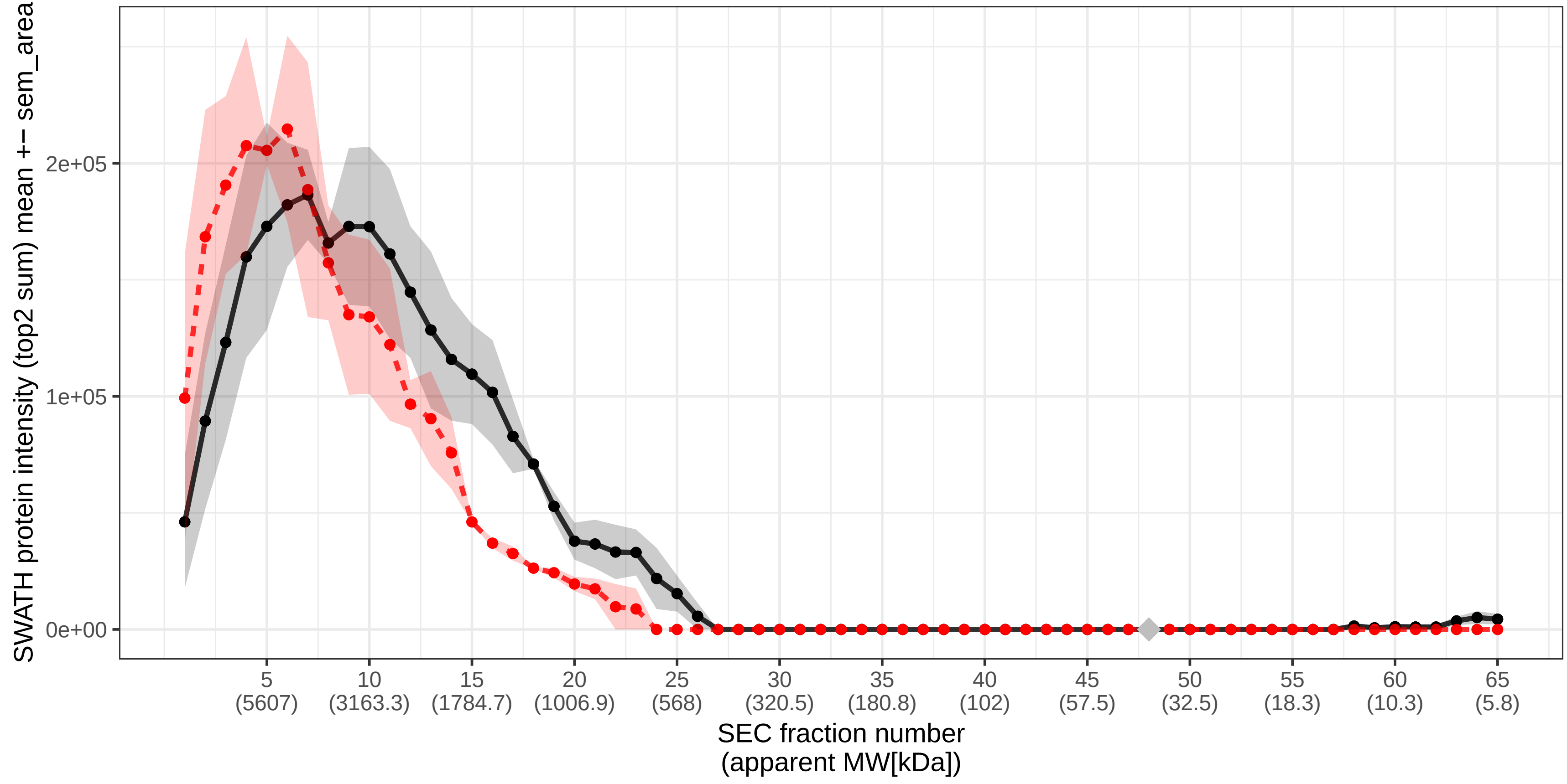

Supplement: Data S1. SEC-SWATH-MS Protein Chromatograms, Related to Figure 1 [file mmc6.zip › SECchrom_Q9NZJ7_MTCH1_HUMAN_MTCH1_PSAP_CGI-64_UNQ1871_PRO43.pdf]
